# Anchorage Performance of an Innovative Assembled Joint with Large-Diameter Steel Bar Grout Lapping in Concrete Reserved Hole

**DOI:** 10.3390/ma18132950

**Published:** 2025-06-22

**Authors:** Qi Chen, Xiaoyong Luo, Chao Deng, Tai Zhou, Xutong Zheng

**Affiliations:** 1 College of Civil Engineering, Central South University, Changsha 410075, China; chenqi970604@163.com (Q.C.); dcxtuseu@163.com (C.D.); csuzhoutai@163.com (T.Z.); 234811062@csu.edu.cn (X.Z.); 2 Engineering Technology Research Center for Prefabricated Construction Industrialization of Hunan Province, Changsha 410075, China

**Keywords:** anchorage performance, assembled joint, high-strength grouting material, large-diameter steel bar, anchorage length, pull-out test

## Abstract

To investigate the anchorage performance of an innovative assembled joint with large-diameter steel bar grout lapping in a concrete reserved hole, the effects of anchorage length and high-strength grouting material types on the failure mode, load–displacement curve, ultimate bond strength and strain variation were analyzed through the pull-out tests of 15 specimens. On this basis, the calculation formulae of critical and ultimate anchorage length were established and the applicability was verified, and then the recommended value of minimum anchorage length was provided. The results showed that the failure modes included splitting-steel bar pull-out failure and UHPC-concrete interface failure. With the increase in anchorage length, the bond strength showed a trend of increasing first and then decreasing. Increasing the grouting material strength can effectively improve the bond performance. When the anchored steel bar is HRB400 with a diameter not less than 20 mm, the recommended minimum anchorage length is 15.0*d*~18.3*d*. When the grouting material strength is larger than or equal to 100 MPa, the anchorage length should not be less than 15.0*d*.

## 1. Introduction

Compared with traditional construction technology, prefabricated structures have been rapidly developed and widely used worldwide due to their advantages, including low energy consumption, high construction efficiency and less on-site work [1,2,3,4,5]. The key technology of prefabricated reinforced concrete structures is the reliable connection between the precast components [6,7,8]. At present, the joint connection methods used in prefabricated reinforced concrete structures mainly include bolt connection, grouting sleeve connection and grout splicing connection [9,10,11]. Bolt connection is a method of connecting precast components together by high-strength bolts and embedded steel plates, which is a dry connection method [12]. The grouting sleeve connection is to pour the high fluidity grouting material into the space between the steel sleeve and the steel bar to achieve the anchorage connection [13,14,15]. Grout splicing connection is to insert the steel bar into the reserved hole at the corresponding position of the precast components, and then pour the high-strength non-shrinkage grouting material into the space between the steel bar and the reserved hole to play an anchoring role [16,17]. It mainly includes two forms: grouted spiral-confined lap connection [16,18] and metal corrugated pipe grout splicing connection [17,19,20]. How to improve the connection performance is a crucial and difficult problem to ensure the behavior of prefabricated reinforced concrete structures [21,22,23,24].

The existing research found that the bolt connection has problems, such as difficulty in controlling the pre-tightening force, requiring a larger operating space and a great number of connectors, making the construction site inconvenient to manage [25]. The grouting sleeve connection has problems such as difficult positioning, high construction accuracy requirements and uncompacted grouting [26,27,28]. However, the grout splicing connection has been extensively applied in prefabricated reinforced concrete structures due to the superiorities of expedient construction, high grouting quality, strong connection and wide application field [29,30]. Relevant researchers have explored the connection performance of spiral stirrup grout splicing connection and metal corrugated pipe grout splicing connection. Chen et al. [31] found that there were three kinds of damage modes that occurred in 52 specimens, and the bond strength showed a trend of increasing with the increase in the overlap length. Wang et al. [32] found that when *D*/*d* is 2.5~3.5, the bond behavior is excellent, and the suggested anchoring length is 10~15*d*. Wan et al. [16] conducted 18 groups of tests and concluded that the specimens embedded with steel bars with a diameter of 25 or 28 mm would not experience pull-out failure, and the anchorage performance was good. Zhou et al. [33] investigated the stainless reinforcing bars in the grouted duct and found that the longer the embedded length of stainless reinforcing bars, the less likely the specimens were to show rebar pull-out failure and concrete splitting failure, and were more prone to fracture failure of the bar. What is more, many researchers have studied the bond behavior of concrete and grout materials [34,35,36]. Tullini et al. [37] designed and manufactured a prefabricated reinforced concrete column with corrugated steel sleeves grouting connection and carried out experimental research. It was found that the damage occurred far away from the joint connection area. Aiamsri et al. [38] investigated the behavior of lap-spliced rebars in UHPC and found that bond strength increased with lap-splice length. Because UHPC had higher compressive strength, it exhibited significantly stronger bond properties. Alkaysi et al. [39] concluded that the bond behavior between UHPC and steel bars showed a large dispersion, and the main influencing factors were nominal diameter, anchorage length, steel fiber volume and casting orientation.

**Figure 1:**
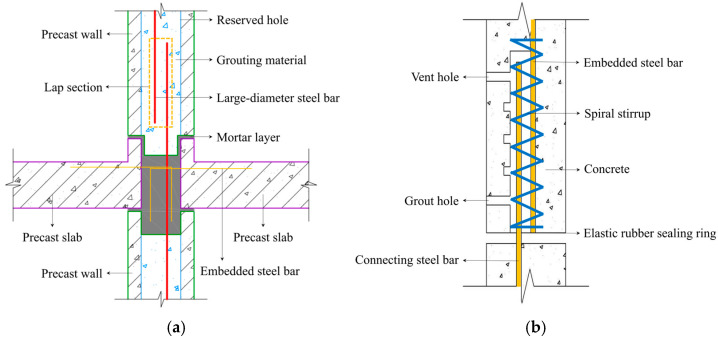
The schematic diagrams of joint: (**a**) large-diameter steel bar grout lapping in reserved hole; (**b**) spiral stirrup grout splicing connection.

In summary, the anchorage performance of the spiral stirrup grout splicing connection and metal corrugated pipe grout splicing connection is excellent, and the main influencing factors are the diameter of the anchored steel bar, the anchorage length, the diameter of the corrugated pipe and the strength of grouting material. However, it is worth noting that the corrugated pipe requires higher fluidity of the grouting material. Otherwise, problems such as hole blockage or grout leakage are prone to occur. On the basis of the above problems, our research group introduced an innovative joint [40], as shown in Figure 1. In the horizontal direction, the special waterproof mortar is used to connect precast components to form a mortar layer. In the vertical direction, the large-diameter steel bars are arranged concentratedly in full-length vertical reserved holes and anchored together with high-strength grouting materials, which can achieve the connection in two directions between the precast walls and the precast slabs. This connection technology does not require a template, and the joint connection and assembly can be realized effectively.

It is well known that the factors affecting the anchorage performance mainly include concrete strength, the thickness of the concrete cover, the diameter of the steel bar, the anchorage length and the position of the steel bar. However, the difference between this paper and the traditional research is that it focuses on the anchorage performance of large-diameter steel bar grout lapping in concrete reserved hole technology proposed by our research group, which involves three materials (concrete, high-strength grouting material and steel bar) and two interfaces.

In view of the fact that the innovative connection technology is suitable for the joint, the concrete strength grade of precast concrete structure in practical engineering is mostly C30 and the thickness of the wall is mostly 200 mm, so the two are not considered as the key factors. In addition, the large-diameter steel bars in the concrete reserved hole of the precast components are often arranged at the center of the hole to ensure effective bonding, so the effect of the position of the steel bar can be ignored. For this paper, the anchorage length and the high-strength grouting material types are the most outstanding factors affecting the anchorage performance. Therefore, the pull-out tests of 15 specimens were conducted to inspect the failure mode, load–displacement curve, ultimate bond strength and strain variation. Finally, the calculation formulae of the critical and the ultimate anchorage length of large-diameter steel bar grout lapping in a concrete reserved hole were established according to the fitting analysis of the experimental observation data, and the recommended value of the minimum anchorage length was given.

## 2. Experimental Program

### 2.1. Test Specimens

**Figure 2:**
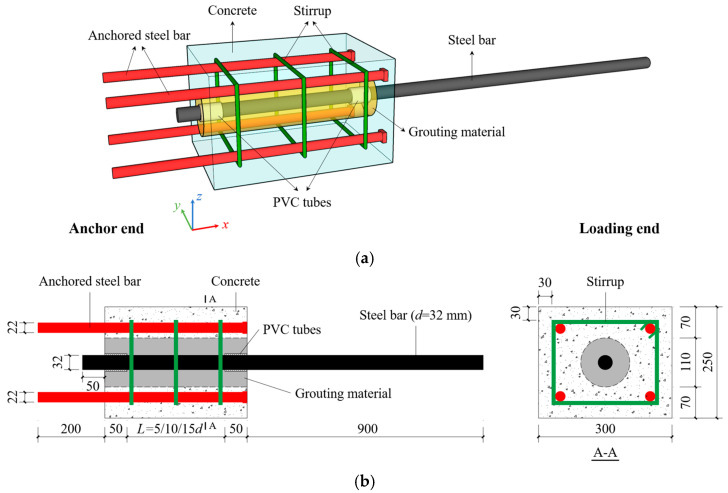
Design of specimens (unit: mm): (**a**) three-dimensional view; (**b**) geometric dimensions and construction.

**Figure 3:**
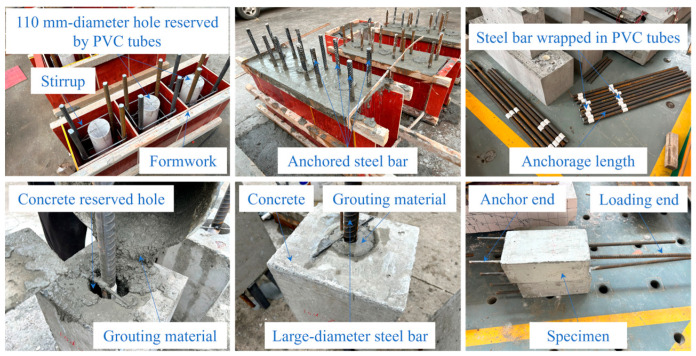
Manufacturing process of specimens.

To identify the failure mode and stress mechanism of the innovative assembled joint veritably, and to examine the anchorage performance of large-diameter steel bar and high-strength grouting material in the concrete reserved hole, five groups of 15 specimens were designed, and the three-dimensional view of the specimen is shown in Figure 2a. The cross-section size is 250 × 300 mm, made of C30 concrete. A 110 mm diameter hole was reserved in the geometric center of the specimen using polyvinyl chloride (PVC) tubes, high-strength grouting material (60~100 MPa) was poured into the reserved hole and the HRB400 grade steel bar with a diameter (*d*) of 32 mm was anchored (called loading end). For the convenience of loading, four HRB400-grade anchored steel bars with an extension length of 200 mm and a diameter of 22 mm were embedded in concrete (called anchor end). To simulate the state of this connection method in precast shear walls more realistically and enhance the lateral restraint of the bond segment, HPB300 grade stirrups (190 × 240 mm, *d* = 8 mm) were configured with a spacing of 150 mm. The research variables are mainly the anchorage length and the grouting material strength. The anchorage length (*L*) is set to 5*d*, 10*d* and 15*d* (160, 320 and 480 mm). For the purpose of reducing the impact of stress concentration on the specimen in the pull-out tests, PVC tubes and polyurethane foam sealing agent were used to isolate the steel bars outside the anchorage section to simulate the unbonded section. The geometric size of the specimens is shown in Figure 2b. The types of high-strength grouting materials are self-compacting concrete (SCC), ultra-high performance concrete (UHPC) and bean-stone grouting material (BSGM). The information on specimens is shown in Table 1, and the manufacturing process is exhibited in Figure 3. The production, curing and grouting process all follow the requirements of the specifications.

### 2.2. Materials

**Figure 4:**
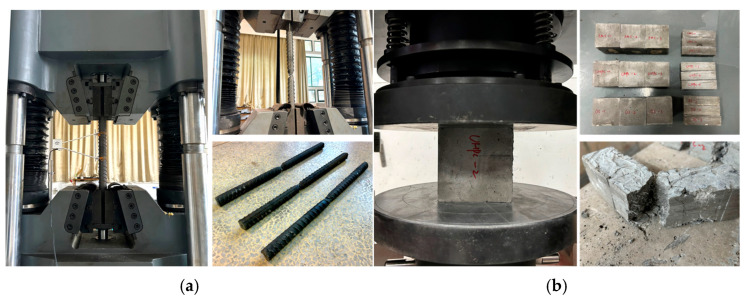
Process of material performance testing: (**a**) material performance testing of steel bar; (**b**) material performance testing of concrete, SCC, UHPC and BSGM.

The concrete strength grade is C30, and six concrete cube specimens are prepared according to GB/T 50081-2019 [41]. The compressive strength test proceeded after 28 days of curing, and the average compressive strength was 33.4 MPa. The mechanical properties of HRB400 with a diameter of 32 mm are shown in Table 2. The design strength grade of SCC is C60; UHPC is mixed with 2% flat-straight copper-plated steel fiber and its design compressive strength is 100 MPa; the content of bean-stone in BSGM is about 10%, and the particle size is 3–10 mm of continuous gradation. Three cubic specimens were reserved for each grouting material according to GB/T 50081-2019 [41], and the compressive strength tests proceeded. The process of material performance testing is shown in Figure 4, and the test results are listed in Table 3.

### 2.3. Pull-Out Tests

**Figure 5:**
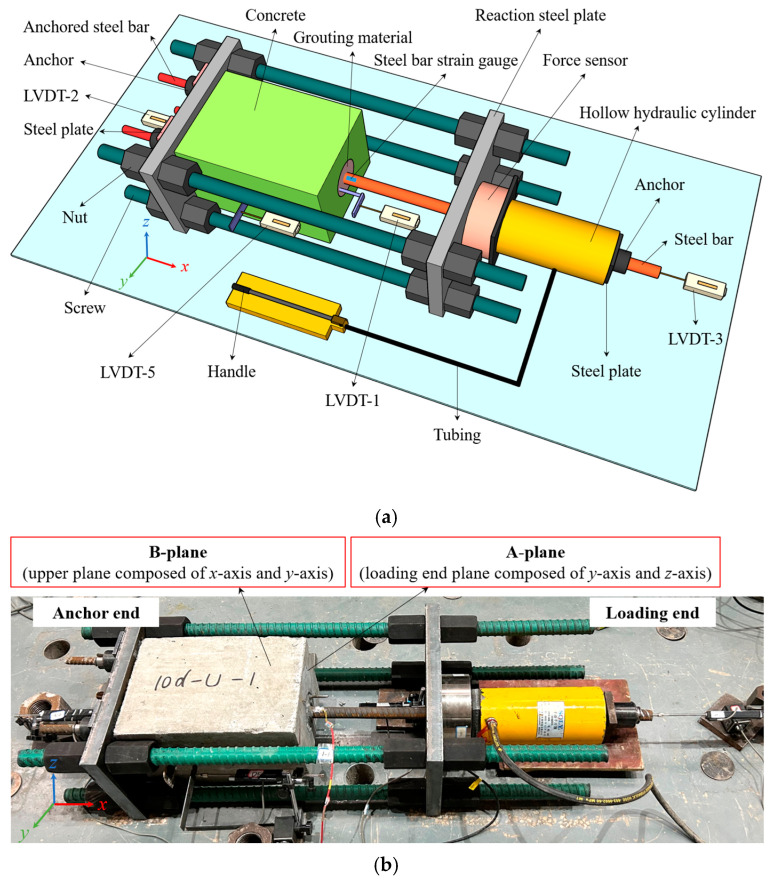
Pull-out test system: (**a**) three-dimensional view; (**b**) photographic image.

The tests were conducted under dry and normal temperature conditions, and the pull-out test system is shown in Figure 5. A reaction frame system with two ends that can be freely adjusted was designed, consisting of four 40 mm diameter screws, 16 matching nuts and two 40 mm thickness steel plates. A 60 t hollow hydraulic cylinder was used to apply the load in the pull-out test, and the tensile force was measured by a wheel-shaped force sensor. Four linear variable displacement transducers (LVDTs) were arranged to know the slip of the loading end and free end, the grouting material and the specimen relative to the ground. In addition, a steel bar strain gauge was arranged at the root of the steel bar at the loading end (near the surface of the specimen) to measure the strain change during the pull-out test.

The bond strength and relative slip can be calculated by Equations (1)–(4) [42]:

(1) $$\tau =\frac{F}{\pi dL}$$

(2) $$\Delta S_{L}=\frac{Fl_{w}}{1000E_{s}A_{s}}$$

(3) $$S_{L}=S_{L}^{\prime }-\Delta S_{L}$$

(4) $$S=\frac{1}{2}\left(S_{L}+S_{F}\right)$$

where *τ* is the ultimate bond strength, *F* is the ultimate tensile force of the steel bar, Δ*S*_L_ is the elongation deformation of the steel bar, *l*_w_ is the free extension length of the steel bar, *E*_s_ is elastic modulus, *A*_s_ is the cross-sectional area of the steel bar, *S*_L_ is the slip of loading end, *S*_L_^′^ is the slip read by LVDT-3, *S* is the relative slip and *S*_F_ is the slip of free end.

## 3. Results and Discussion

### 3.1. Failure Mode

#### 3.1.1. Test Phenomena

**Figure 6:**
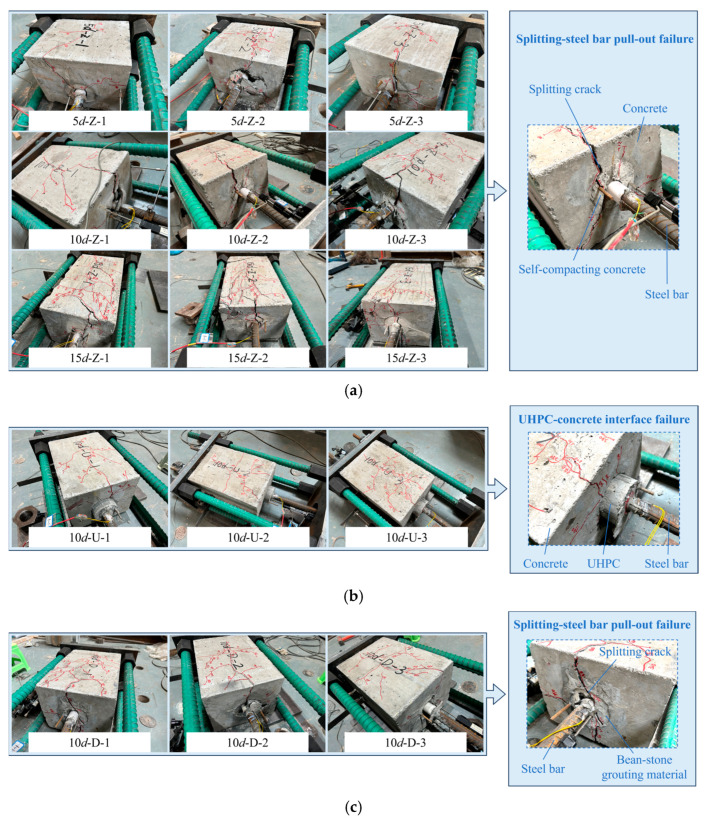
Failure mode of specimens: (**a**) 5*d*/10*d*/15*d*-Z; (**b**) 10*d*-U; (**c**) 10*d*-D.

The failure mode basically included two forms: splitting-steel bar pull-out failure and UHPC-concrete interface failure, as shown in Figure 6. It can be seen that 5*d*-Z,10*d*-Z,15*d*-Z and 10*d*-D showed splitting-steel bar pull-out failure, the steel bar was slowly pulled out along with the joint splitting of concrete and SCC or BSGM. While 10*d*-U showed UHPC-concrete interface failure, that is, the UHPC cylinder and concrete remained intact, only shear failure occurred at the interface without any relative slip between the steel bar and UHPC. The definitions of the A-plane and the B-plane are shown in Figure 5.

(a) Splitting-steel bar pull-out failure (SPF)

When the grouting material was SCC, 5*d*-Z, 10*d*-Z and 15*d*-Z all showed splitting-steel bar pull-out failure. Taking 10*d*-Z as an example, the failure process was described as follows: when loaded to 170 kN, SCC and concrete on the A-plane cracked together and extended to the B-plane. As the load increased, there were more and more splitting cracks on the A-plane, which developed from the steel bar to the peripheral areas. When the load reached 190 kN, cracks perpendicular to the steel bar occurred on the B-plane, and they continued to extend and expand. At this time, cracks parallel to the steel bar also developed and penetrated, becoming dense. When loaded to 260 kN, the initial crack was significantly wider, about 1.6 mm. As the load continued to increase until 320 kN, the specimen was split and accompanied by a crackling sound. The steel bar and SCC were debonded, and the relative slip began to occur between the two. At this point, the width of the initial crack reached 7.5 mm. Subsequently, the steel bar was pulled out and the relative slip increased accordingly, while the bond behavior between SCC and concrete was good. The failure modes of 5*d*-Z and 15*d*-Z were similar to the above phenomena.

When the grouting material was BSGM, the failure mode was also splitting-steel bar pull-out failure. When loaded to 200 kN, the concrete and BSGM cracked together on the A-plane and extended to the B-plane. Subsequently, cracks perpendicular to the loading direction appeared at about half of the B-plane and quickly penetrated. With the gradual increase in load, the cracks in both directions were developed, and the cracks along the direction of steel bars were more pronounced and densely. When the load reached 280 kN, the cracks on the B-plane showed a trend of extending towards the corners of the loading end and continued to extend and connect. In this process, the main cracks expanded and the width became increasingly apparent. When loaded to 350 kN, the specimen suddenly split along the main crack, making a crackling sound and the main crack became visibly wider, which is about 3.2 mm. At this time, the concrete and BSGM were slightly peeled off. As the test continued, the load no longer increased.

(b) UHPC-concrete interface failure (UCF)

When the grouting material was UHPC, the failure mode showed UHPC-concrete interface failure. When loaded to 200 kN, the incipient crack appeared on the A-plane and developed into the B-plane. And the cracks parallel to the steel bar on the B-plane extended towards the anchor end, and it is worth noting that micro-cracks appeared at the junction of the steel bar and UHPC. When loaded to 260 kN, cracks occurred in the 1/2 range of the B-plane and continued to extend. When the load was loaded to more than 310 kN, the cracks on the B-plane showed a trend of gradually extending towards the corners of the loading end. At the moment, the width of the major crack was 1.1 mm, and the cracks perpendicular to the steel bar were basically connected. When the load was about 340 kN, the load no longer increased, and the specimen was damaged and accompanied by a loud sound. At this time, UHPC was separated from concrete and shear failure occurred. The UHPC cylinder and concrete were basically intact with slight peeling off at the interface area. It was worth noting that the steel bar was well bonded to the surrounding UHPC and there was no relative slip. As the test continued, the UHPC cylinder was pulled out of the concrete visibly, and the slip increased accordingly.

By comparing the failure process of specimens with different grouting materials and different anchorage lengths, it was found that for specimens with SCC as grouting material, the steel bars of 5*d*-Z and 10*d*-Z did not yield, while the steel bars of 15*d*-Z yielded before pulling out. The reason was that with the increase in the anchorage length, the bond stress increased continuously. When the anchorage length was the same and the grouting materials were different, the failure process and failure mode of 10*d*-Z and 10*d*-D were similar, but the difference was that the surface cracks of 10*d*-D were more densely distributed, and more BSGM were brought out when the anchorage steel bars were pulled out. In addition, the cracks of 10*d*-U showed a relatively sparse distribution and the crack width was small. The failure mode was characterized by the debonding of the interface between UHPC and concrete. Due to the bond–slip failure between UHPC and concrete, the surface of the UHPC cylinder showed obvious friction marks. The reason was that the strength of UHPC was significantly higher than that of SCC and BSGM. During the test, UHPC can always maintain good bond behavior with large-diameter steel bars, which ensures the effective restraint of UHPC on steel bars. Therefore, the interface between concrete and UHPC reached the ultimate bearing capacity before the UHPC was sheared by large-diameter steel bars.

#### 3.1.2. Internal Damage

**Figure 7:**
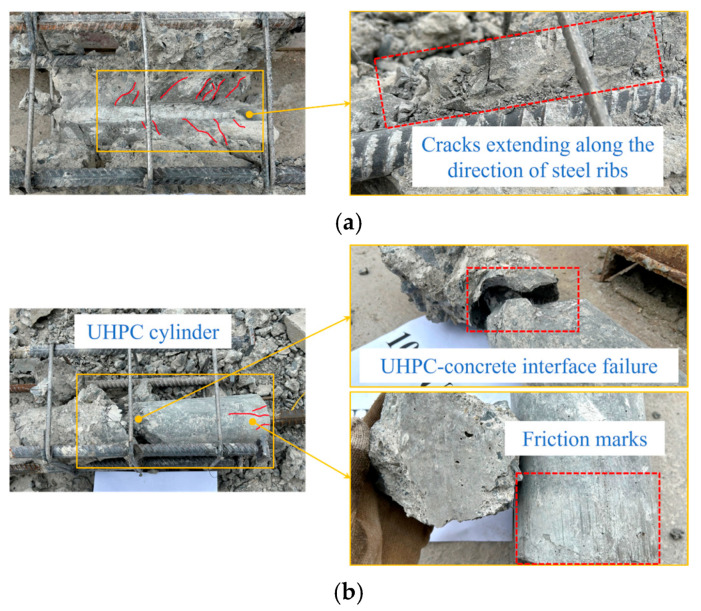

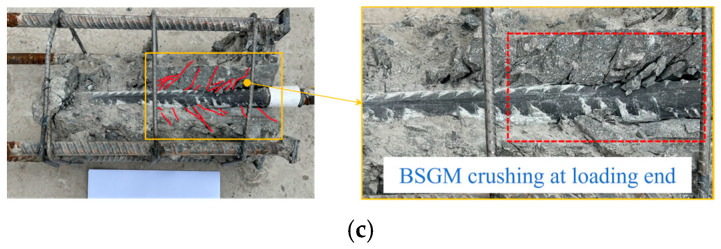
Internal damage of specimens: (**a**) 10*d*-Z; (**b**) 10*d*-U; (**c**) 10*d*-D.

Next, 10*d*-Z, 10*d*-U and 10*d*-D were broken to further observe the internal damage, as shown in Figure 7. It can be found that the peripheral concrete of 10*d*-Z and 10*d*-D maintained a good bond behavior with SCC and BSGM. Some SCC and BSGM near the loading end were sheared and crushed to form a wedge and pulled out together with the anchorage steel bar, while there was basically no relative slip at the interface between other parts and concrete. What is more, cracks along the direction of steel ribs were distributed on both SCC and BSGM, but it was worth noting that the cracks in 10*d*-D were more dense, and the crushing and spalling of the loading end were more serious. In addition, it can be seen that there were obvious SCC and BSGM fragments attached between the ribs at the loading end, indicating that the steel bar slipped relative to SCC and BSGM. For 10*d*-U, the UHPC cylinder was broken into two parts, and the position was about half of the specimen (the section of the cylinder was wedge-shaped). The UHPC cylinder at the loading end was bonded to the anchorage steel bar, and its surface was smooth with some friction marks and distributed with a certain number of small cracks, while the UHPC cylinder at the free end was always bonded to the surrounding concrete.

### 3.2. Load–Displacement Curves

**Figure 8:**
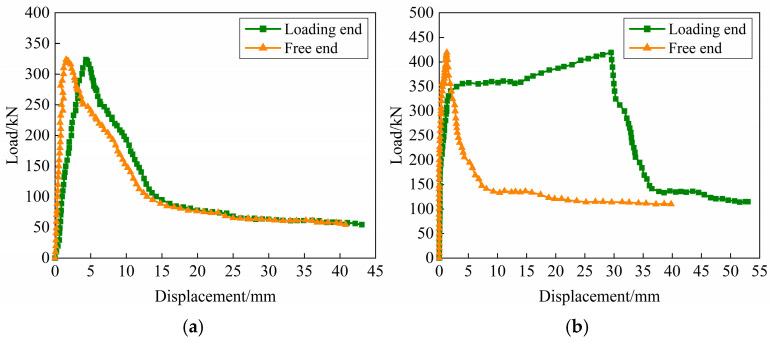
Load–displacement curves at loading end and free end: (**a**) 10*d*-Z-3; (**b**) 15*d*-Z-3.

The test results are summarized in Table 4. According to Section 3.1, it is known that the yield of the steel bar in 15*d*-Z occurs before the splitting-steel bar pull-out failure. Therefore, taking 10*d*-Z-3 with steel bar not yielding and 15*d*-Z-3 with steel bar yielding as examples, the load–displacement curves are exhibited in Figure 8. Among them, the loading end displacement includes the tensile deformation of the steel bar, the sliding of the anchors and the sliding of the steel bar. The stress of the steel bar can be reflected by the tension force of the loading end, which can judge whether the steel bar reached yield, while the displacement at the free end directly reflects the slip of the steel bar, which can roughly represent the bond behavior. From Figure 8a,b, it can be known that the bond behavior of 10*d*-Z-3 fails before the steel bar yields, while the load–displacement curve of 15*d*-Z-3 is completely different, that is, the bond failure shows after the steel bar yields.

What is more, the steel bar at the loading end is mainly subjected to tensile elongation deformation in the initial stage of loading, and there is basically no displacement at the free end. The bond behavior of this stage is generally provided by the chemical adhesive force. As the load increases, some cracks appear and extend due to the extrusion of the steel bar ribs. At this time, the slip shows at the free end and gradually grows. It is worth noting that due to the elastic deformation of the steel bar at the loading end, the displacement keeps becoming larger than that of the free end. The bond behavior is provided by friction force and mechanical bite force at this stage. When the slip develops continuously, the tensile force decreases rapidly, while the displacement increases greatly. The grouting material is sheared by ribs and gradually develops to the whole anchorage length, and then the steel bar begins to be pulled out tardily. Subsequently, the specimen enters the residual stage with a sharp increase in displacement and a steady tensile force. And the bonding is mainly provided by the friction force. In addition, the free end displacement shows a trend of gradually approaching the loading end in the residual stage, which may be owing to partially restoring the elastic deformation of the steel bar at the loading end.

**Figure 9:**
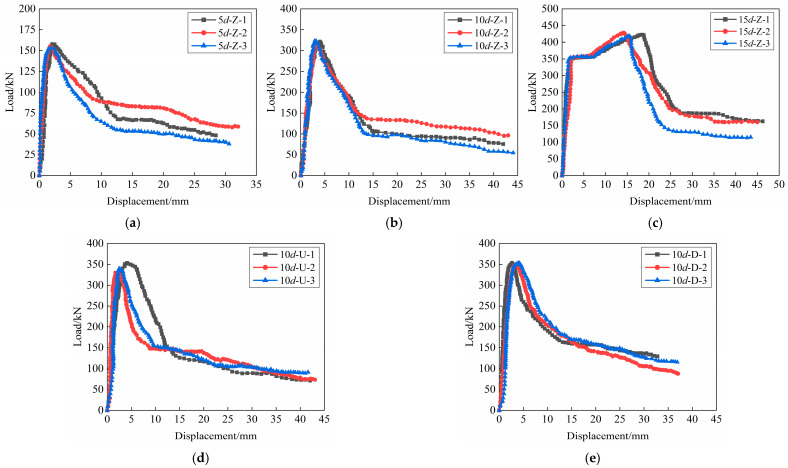
Load–displacement curves: (**a**) 5*d*-Z; (**b**) 10*d*-Z; (**c**) 15*d*-Z; (**d**) 10*d*-U; (**e**) 10*d*-D.

The load–displacement curves of 5*d*-Z, 10*d*-Z, 15*d*-Z, 10*d*-U and 10*d*-D are shown in Figure 9. It can be observed from Figure 9a–c that the anchorage length has a significant effect on load–displacement curve. Comparing 5*d*-Z and 10*d*-Z, it is found that the peak load of 10*d*-Z is significantly higher than that of 5*d*-Z (the peak loads are 155.30 kN and 319.03 kN, respectively). For 15*d*-Z, the variation law is completely different, showing a trend of steel bars yielding first and then strengthening (the peak load is 423.43 kN). This illustrates that the anchorage length is long enough to resist the external load, and the yield of the steel bar occurs before the splitting-pull-out failure. When the grouting material is SCC, the bond stress accumulates in a longer anchorage length and is unevenly distributed along the steel bar, which further increases the failure load.

Comparing Figure 9b,d,e, it can be seen that the effect of the high-strength grouting material types on the load–displacement curve is not remarkable. Compared with 10*d*-Z, the peak bearing capacity of 10*d*-U and 10*d*-D is slightly higher, which are 319.03 kN, 341.07 kN and 352.40 kN, respectively. This is because the compressive strength of SCC is slenderly lower than that of UHPC and BSGM, which makes the peak bearing capacity of specimens with SCC as grouting material slightly lower. Under the increasing load, the interface is gradually sheared and damaged, and the curves gradually enter the residual stage. The residual load of the three different grouting material specimens is basically stable at around 120 kN, which is about 35% of the peak load.

### 3.3. Bond Strength

**Figure 10:**
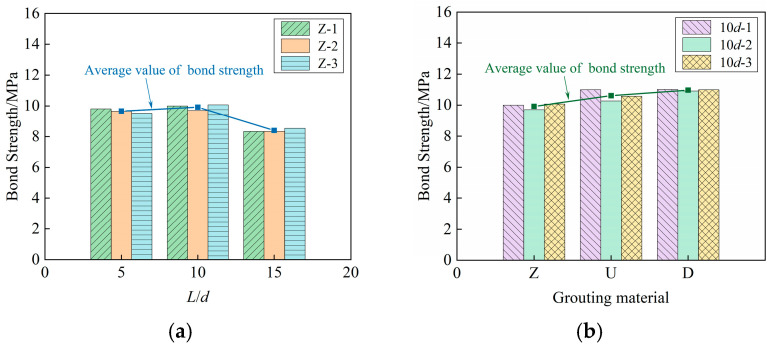
Variation curves of bond strength: (**a**) effect of anchorage length; (**b**) effect of grouting material.

The variation curve of the effect of anchorage length on bond strength is shown in Figure 10a. It can be observed that the bond strength generally shows a slight increase followed by a decrease with the increase in anchorage length. The bond strength of 10*d*-Z increased by about 2.69% compared with that of 5*d*-Z, while the bond strength of 15*d*-Z decreased by about 13.04% compared with that of 5*d*-Z. This indicates that within a certain range, the increase in anchorage length can slightly enhance the bond behavior of the specimens, but it is not significant. When the anchorage length is too large, the distribution of bond strength is uneven. Under the external load, the bond stress at the loading end is relatively large. When the free end reaches the ultimate bond strength, the slip failure occurs at the loading end; consequently, the bond strength diminishes.

Figure 10b shows the effect of SCC, UHPC and BSGM on bond strength under the same anchorage length. It can be observed that the bond strength of specimens with SCC as grouting material is the smallest, followed by UHPC, and the BSGM is the largest, which are 9.92 MPa, 10.60 MPa and 10.96 MPa, respectively. The bond strength of 10*d*-D is about 10.48% higher than that of 10*d*-Z, indicating that the bond strength is positively correlated with the grouting material strength. The main reason is that the higher strength can delay the cracking of grouting material in front of the steel bar ribs under shear force.

### 3.4. Strain of the Steel Bar

**Figure 11:**
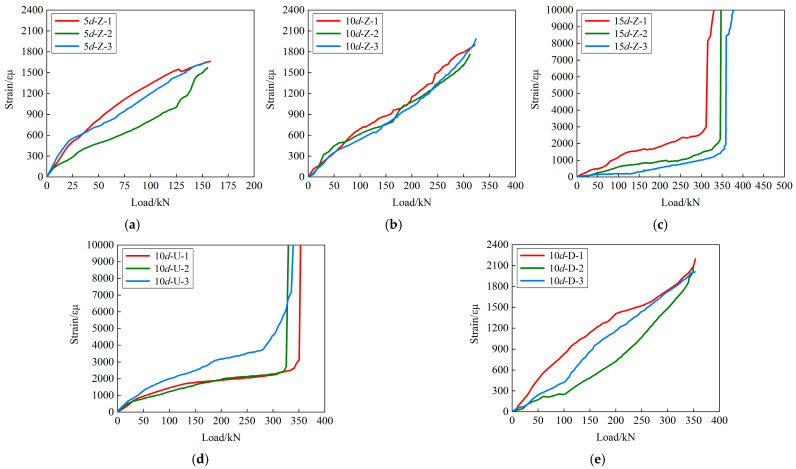
Variation curves of strain: (**a**) 5*d*-Z; (**b**) 10*d*-Z; (**c**) 15*d*-Z; (**d**) 10*d*-U; (**e**) 10*d*-D.

The variation curve of strain with load (before reaching the peak load) is shown in Figure 11. It can be seen that the strains of 5*d*-Z, 10*d*-Z and 10*d*-D basically show a linear increasing trend with load. At the peak load, their strains are 1627.4 εμ, 1883.0 εμ and 2095.2 εμ, respectively, which have not reached yield. However, when the peak load is reached, the strains of 15*d*-Z and 10*d*-U increase suddenly and reach the yield strain. For 15*d*-Z, the anchorage length is sufficient to resist the external pull-out load, so that the steel bar first yields and then the splitting-steel bar pull-out failure is shown. For 10*d*-U, the addition of steel fiber improves the tensile strength and toughness, thereby enhancing the bonding between UHPC and the steel bar. Therefore, 10*d*-U has experienced UHPC-concrete interface failure after the peripheral concrete split and the steel bar yields. In this process, the bond behavior between UHPC and steel bar is always satisfactory. In addition, the strain of 10*d*-U-3 is significantly higher than the other two specimens. The reason may be that the fabrication error leads to the inclination of the anchored steel bar, which leads to an abnormal increase in the strain.

## 4. Calculation of Anchorage Length

### 4.1. Critical Anchorage Length and Ultimate Anchorage Length

At present, many researchers have studied the anchorage performance of steel bar grout splicing and given the recommended anchorage length [16,43,44,45,46]. It is well known that the diameter of the steel bar, anchorage length, grouting material strength and diameter of metal corrugated pipes are the main influencing parameters. The innovative assembled joint shown in Figure 1 is similar to the existing research, but the difference is that the diameter of the steel bar and concrete reserved hole is larger, and there are no embedded metal corrugated pipes. For the anchorage performance of this innovative connection technology, the key influencing factors are the diameter of the steel bar, anchorage length and grouting material.

For this reason, in view of the characteristics of this innovative assembled joint proposed by our research group, Section 4 focuses on the calculation method of anchorage length. This connection technology is mainly used in the assembly between precast concrete walls and precast concrete slabs, and the common concrete strength grade of precast components in practical engineering is C30 (that is, the peripheral concrete strength grade of the specimen is C30). Therefore, the concrete strength grade is not the main consideration, which can be ignored in this paper. In addition, in order to ensure the reliability of the joint connection, the large-diameter steel bars should be arranged in the center of the concrete reserved hole, so the effect of the steel bar position can be ignored.

Based on the test results of the above 15 pull-out specimens, taking the diameter of the steel bar and grouting material as the main influencing factors, the calculation formula of anchorage length is established by regression fitting and theoretical derivation. When specimen failure and steel bar yield occur simultaneously, it is the critical anchorage length, that is, the minimum length to ensure that the anchorage failure will not occur before the steel bar yield. When the ultimate bond strength is equivalent to the ultimate tensile strength, it is the ultimate anchorage length, that is, the minimum length to ensure that no anchorage failure will occur before the steel bars reach ultimate strength.

**Figure 12:**
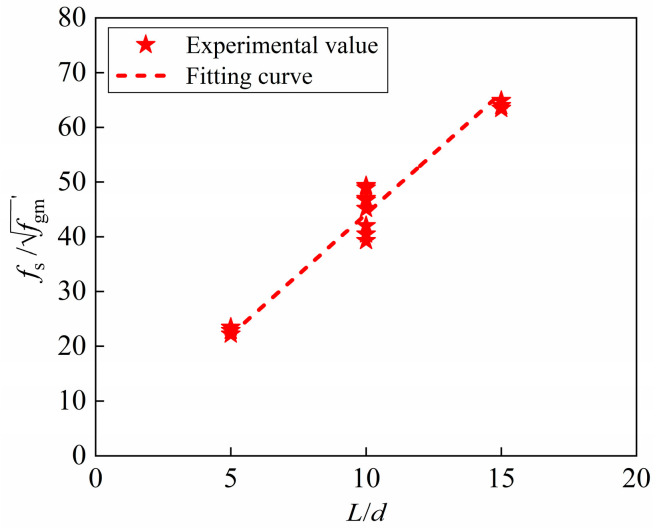
Fitting curve.

The corresponding relationship between the relative ultimate bond strength and the relative anchorage length can be built based on the fitting of the pull-out test data. Since three different grouting materials (SCC, UHPC and BSGM) are used, the ultimate bond strength is normalized by the grouting material strength to more intuitively reflect its relationship with the relative anchorage length, as shown in Figure 12. The relative ultimate bond strength shows an increasing trend as the relative anchorage length, and there is a significant linear relationship between them. The statistical metrics of the fitting analysis are as follows: slope *k* = 4.43, intercept *b* = 3.35 and coefficient of determination *R*^2^ = 0.946. Among them, the coefficient of determination is an important statistic that reflects the goodness of fitting. Since *R*^2^ = 0.946 is very close to 1, the fitting analysis results are reliable and satisfactory.

From the analysis of Section 3.4, it can be known that the steel bars of 15*d*-Z and 10*d*-U have reached yield before the bond failure, so the critical anchorage length depends on the correlation coefficient, that is, the slope of the curve. The linear relationship can be expressed by Equation (5):

(5) $$\frac{f_{s}}{\sqrt{f_{\mathrm{gm}}^{\prime }}}=k\cdot \frac{l_{\mathrm{cr}}}{d}$$

where *l*_cr_ is the critical anchorage length, *f*_s_ is the stress of the steel bar, *k* is the correlation coefficient and $f_{\mathrm{gm}}^{\prime }$ is the measured value of grouting material strength.

Based on Equation (5), the calculation formula of critical anchorage length can be provided, as shown in Equation (6):

(6) $$l_{\mathrm{cr}}=\frac{f_{s}\cdot d}{k\sqrt{f_{\mathrm{gm}}^{\prime }}}$$

According to the fitting regression analysis of Figure 12, the correlation coefficient *k* is 4.43. Substituting *k* into Equation (6), the critical anchorage length can be derived as Equation (7):

(7) $$l_{\mathrm{cr}}=\frac{f_{s}\cdot d}{4.43\sqrt{f_{\mathrm{gm}}^{\prime }}}$$

Since there is a certain degree of discreteness in the mechanical properties of the steel bar, to guarantee that the steel bar can give sufficient play to its mechanical properties, 1.20 is taken as the amplification factor of the design value of the tensile strength of steel bar, as shown in Equation (8):

(8) $$l_{\mathrm{cr}}=\frac{f_{y}\cdot d}{3.69\sqrt{f_{\mathrm{gm}}^{\prime }}}$$

Similarly, 1.75 is taken as the amplification factor of the design value of the tensile strength of the steel bar. The ultimate anchorage length is as Equation (9):

(9) $$l_{u}=\frac{f_{y}\cdot d}{2.53\sqrt{f_{\mathrm{gm}}^{\prime }}}$$

where *l*_u_ is the ultimate anchorage length, *f*_y_ is the design value of steel bar tensile strength and $f_{\mathrm{gm}}^{\prime }$ is the measured value of grouting material strength.

The critical and ultimate anchorage lengths calculated by Equations (8) and (9) are shown in Table 5. The relationship between anchorage length and failure mode is as follows:

(1) *L* < *l*_cr_, the steel bars have not reached the yield strength but SPF or UCF has occurred;
(2) *l*_cr_ ≤ *L* < *l*_u_, the steel bars have reached yield strength and SPF or UCF has occurred;
(3) *L* ≥ *l*_u_, the steel bars have reached ultimate tensile strength and broken.

From the above analysis, when SCC is grouted, the anchorage lengths of 5*d*-Z and 10*d*-Z are both less than 11.9*d*, which is consistent with the description of (1). While the anchorage length of 15*d*-Z is greater than 11.9*d* and less than 17.3*d*, which is consistent with the description of (2). When UHPC is grouted, the anchorage length of 10*d*-U is greater than 9.3*d* and less than 13.6*d*, which is consistent with the description of (2). When BSGM is grouted, the anchorage length of 10*d*-D is less than 10.9*d*, which is consistent with the description of (1). In summary, the calculated critical and ultimate anchorage lengths are in good agreement with the failure mode (Section 3.1) and steel bar strain (Section 3.4), which verifies the applicability and reliability of the formulae.

Based on the test results, it can be known that there are two failure modes of the specimens in this paper: splitting-steel bar pull-out failure and UHPC pull-out failure. However, it can be seen through the above discussion that only when the steel bars have reached ultimate tensile strength and broken is the anchorage performance of large-diameter steel bar grout lapping in concrete reserved holes is absolutely reliable. Therefore, the calculation formula of the minimum anchorage length is proposed when the strength of the grouting material is 60~100 MPa and the diameter of HRB400 is not less than 20 mm. The recommended value of the minimum anchorage length calculated by Equation (10) is shown in Table 6.

(10) $$l_{\min}=\frac{f_{y}\cdot d}{2.53\sqrt{f_{\mathrm{gm}}}}$$

where *l*_min_ is the minimum anchorage length, *f*_y_ is the design value of steel bar tensile strength, *f*_gm_ is the cube compressive strength of grouting material, in order to make the recommended value have a certain safety reserve, when *f*_gm_ ≥ 90 MPa, *f*_gm_ = 90 MPa.

For the connection technology of the precast components with the grouting material strength is 60~100 MPa, with HRB400 steel bars and not less than 20 mm in diameter, when the reserved hole diameter is 110 mm, the recommended minimum anchorage length is 15.0*d*~18.3*d*.

### 4.2. Comparison of Minimum Anchorage Length

Refer to GB50010-2010 [47], wherein the formula of the fundamental anchorage length is demonstrated as follows:

(11) $$l_{a}=\alpha \frac{f_{y}}{f_{t}}d$$

where *l*_a_ is the anchorage length, *α* is the shape coefficient of the steel bar (the deformed steel bar with ribs is taken as 0.14), *f*_y_ is the design value of steel bar tensile strength and *f*_t_ is the design value of concrete axial tensile strength.

Refer to ACI318-14 [48], wherein the anchorage length of steel bars No. 7 (diameter is 22 mm) and above is calculated using the following formula:

(12) $$l_{a}=\frac{f_{y}\psi_{t}\psi_{e}}{20\lambda \sqrt{f_{c}^{\prime }}}d$$

where *ψ*_t_ and *ψ*_e_ are the position coefficient and coating coefficient of steel bar (*ψ*_t_ = 1, *ψ*_e_ = 1), *λ* is the coefficient of lightweight aggregate concrete (*λ* = 1) and $f_{c}^{\prime }$ is the compressive strength of concrete.

The recommended value of minimum anchorage length is given in the specifications [47,48] and in this paper is listed in Table 6. It can be observed from Table 6 that the minimum anchorage length required for the innovative assembled joint with large-diameter steel bar grout lapping in concrete reserved hole technology is smaller than the corresponding grade concrete calculated by the formula proposed in the specifications. This shows that the bond behavior between the three grouting materials of SCC, UHPC and BSGM and the steel bar is better than that between concrete of the corresponding strength grade and the steel bar. In general, the constraint effect provided by the surrounding concrete, stirrups and high-strength grouting materials limits the slip of the anchored large-diameter steel bar, thereby improving the anchorage performance and effectively shortening the anchorage length of the steel bar.

## 5. Conclusions

In this paper, the anchorage performance of innovative assembled joint with large-diameter steel bar grout lapping in the concrete reserved hole was studied, and the conclusions can be summarized as follows:

(1) When the grouting material is SCC or BSGM, the failure mode is primarily splitting-steel bar pull-out failure. When the grouting material is UHPC, the failure mode changes to UHPC-concrete interface failure, and the anchorage performance between UHPC and steel bar is good without relative slip.
(2) With the increase in anchorage length, the ultimate bond strength first increases and then decreases. When the anchorage length is 10*d*, the ultimate bond strength is the largest, and the anchorage performance is the best. In addition, the increasing of the grouting material strength can also enhance the ultimate bond strength to a certain extent.
(3) The calculation formulae for the critical and ultimate anchorage length of the innovative assembled joint with large-diameter steel bar grout lapping in concrete reserved holes are proposed. The calculation results are in good agreement with the failure mode and the steel bar strain changes, which verifies the applicability and reliability.
(4) When the anchored steel bar is HRB400 with a diameter not less than 20 mm, the recommended minimum anchorage length is 15.0*d*~18.3*d*. When the grouting material strength exceeds 100 MPa, the minimum anchorage length should not be less than 15.0*d*. The minimum anchorage length given in this paper is smaller than the recommended valve of specifications, indicating that the anchorage performance of the innovative assembled joint is satisfactory.

## Figures and Tables

**Table 1:** Design parameters of specimens.

Specimen	*d*/mm	*L*/mm	Dimension/mm	Grouting Material
5*d*-Z	32	5*d* = 160	250 × 300 × 260	Self-compacting concrete (SCC)
10*d*-Z	32	10*d* = 320	250 × 300 × 420	Self-compacting concrete (SCC)
15*d*-Z	32	15*d* = 480	250 × 300 × 580	Self-compacting concrete (SCC)
10*d*-U	32	10*d* = 320	250 × 300 × 420	Ultra-high performance concrete (UHPC)
10*d*-D	32	10*d* = 320	250 × 300 × 420	Bean-stone grouting material (BSGM)

Note: *d* is the diameter of steel bar, *L* is the anchorage length.

**Table 2:** Mechanical properties of steel bar.

Grade	*d*/mm	Yield Strength/MPa	Tensile Strength/MPa	Elongation/%
HRB400	32	434.60	633.31	30.25
HRB400	32	433.38	632.58	26.63
HRB400	32	430.87	631.93	29.38

Note: *d* is the diameter of steel bar.

**Table 3:** Compressive strength of grouting material.

Grouting Material	Load/kN	*f*_c_/MPa
SCC	711.05	67.55
UHPC	1149.26	109.18
BSGM	836.53	79.47

Note: *f*_c_ is the compressive strength.

**Table 4:** Summary of pull-out test results.

Specimen	*L*/mm	*F*/kN	*F*_avg_/kN	*τ*/MPa	*τ*_avg_/MPa	*S*_L_/mm	*S*_F_/mm	*S*/mm	*S*_avg_/mm	Failure Mode
5*d*-Z-1	160	157.70	155.30	9.81	9.66	2.53	1.63	2.08	1.91	SPF
5*d*-Z-2	160	155.10		9.65		2.30	1.20	1.75		SPF
5*d*-Z-3	160	153.10		9.52		2.40	1.40	1.90		SPF
10*d*-Z-1	320	321.60	319.03	10.00	9.92	6.31	1.69	4.00	3.40	SPF
10*d*-Z-2	320	311.80		9.70		5.20	1.30	3.25		SPF
10*d*-Z-3	320	323.70		10.07		4.35	1.55	2.95		SPF
15*d*-Z-1	480	422.50	423.43	8.33	8.40	/	/	/	/	SPF
15*d*-Z-2	480	428.60		8.33		/	/	/		SPF
15*d*-Z-3	480	419.20		8.54		/	/	/		SPF
10*d*-U-1	320	353.20	341.07	10.98	10.60	6.23	1.95	4.09	2.75	UCF
10*d*-U-2	320	330.00		10.26		3.15	0.25	1.70		UCF
10*d*-U-3	320	340.00		10.57		3.68	1.25	2.47		UCF
10*d*-D-1	320	353.60	352.40	11.00	10.96	4.06	1.24	2.65	3.48	SPF
10*d*-D-2	320	350.40		10.90		5.50	1.90	3.70		SPF
10*d*-D-3	320	353.20		10.98		6.31	1.89	4.10		SPF

Note: *F* is the tensile force, *F*_avg_ is the average value of the tensile force, *τ* is the bond strength, calculated by Equation (1), *τ*_avg_ is the average value of the bond strength, *S*_L_ is the slip of loading end, *S*_F_ is the slip of free end, *S* is the relative slip, calculated by Equations (2)–(4), *S*_avg_ is the average of the relative slip.

**Table 5:** Summary of minimum anchorage length.

Grouting Material	$f_{\mathrm{gm}}^{\prime }$/MPa	*l*_cr_/mm	*l*_u_/mm
SCC	67.55	11.9*d*	17.3*d*
UHPC	109.18	9.3*d*	13.6*d*
BSGM	79.47	10.9*d*	16.0*d*

Note: $f_{\mathrm{gm}}^{\prime }$ is the measured value of grouting material strength, *l*_cr_ is the critical anchorage length, *l*_u_ is the ultimate anchorage length.

**Table 6:** Comparison of minimum anchorage length.

Category	Minimum Anchorage Length/mm				
	*f* = 60 MPa	*f* = 70 MPa	*f* = 80 MPa	*f* = 90 MPa	*f* = 100 MPa
This paper	18.3*d*	17.0*d*	15.9*d*	15.0*d*	15.0*d*
GB50010-2010 [47]	24.7*d*	23.6*d*	22.7*d*	/	/
ACI318-14 [48]	28.0*d*	25.9*d*	24.2*d*	22.9*d*	21.7*d*

Note: *f* is the strength grade of grouting material.

## Data Availability

The original contributions presented in this study are included in the article. Further inquiries can be directed to the corresponding authors.

## Abbreviations

The following abbreviations/nomenclature are used in this manuscript:

**Abbreviations**	
SCC	Self-compacting concrete
UHPC	Ultra-high performance concrete
BSGM	Bean-stone grouting material
PVC	Polyvinyl chloride
LVDT	Linear variable displacement transducer
**Nomenclature**	
*d*	diameter of steel bar
*L*	anchorage length
*f*_c_	compressive strength
*τ*	ultimate bond strength
*τ*_avg_	average value of ultimate bond strength
*F*	ultimate tensile force of steel bar
*F*_avg_	average value of ultimate tensile force of steel bar
Δ*S*_L_	elongation deformation of steel bar
*l*_w_	free extension length of steel bar
*E*_s_	elastic modulus
*A*_s_	cross-sectional area of steel bar
*S*_L_	slip of loading end
$S_{L}^{\prime }$	slip read by LVDT-3
*S*	relative slip
*S*_avg_	average value of relative slip
*S*_F_	slip of free end
*l*_cr_	critical anchorage length
*k*	correlation coefficient
$f_{\mathrm{gm}}^{\prime }$	measured value of grouting material strength
*l*_u_	ultimate anchorage length
*l*_min_	minimum anchorage length
*f*_gm_	cube compressive strength of grouting material
*α*	shape coefficient of steel bar
*f*_t_	tensile strength
*ψ*_t_	position coefficient of steel bar
*ψ*_e_	coating coefficient of steel bar
*λ*	coefficient of lightweight aggregate concrete

## References

[1] Guri M., Brzev S., Lluka D. (2021). Performance of Prefabricated Large Panel Reinforced Concrete Buildings in the November 2019 Albania Earthquake. J. Earthq. Eng..

[2] Romero A., Moustafa M. (2025). Shake table tests of economical precast ultra-high performance concrete bridge piers with different fiber types and seismic joint materials. Eng. Struct..

[3] Sun C., Zhuang M.L., Wang Z., Chen B., Gao L., Qiao Y., Zhu H., Zhang W., Yang J., Yu C. (2020). Experimental investigation on the seismic performance of prefabricated fiber-reinforced concrete beam-column joints using grouted sleeve connections. Struct. Concr..

[4] Esmaeili J., Khoshkanabi S.P., Andalibi K., Kasaei J. (2023). An innovative method for improving the cyclic performance of concrete beams retrofitted with prefabricated basalt-textile-reinforced ultra-high performance concrete. Structures.

[5] Sui L., Fan S., Huang Z., Zhang W., Zhou Y., Ye J. (2020). Load transfer mechanism of an unwelded, unbolted, grouted connection for prefabricated square tubular columns under axial loads. Eng. Struct..

[6] Wu C., Liu J., Shi W. (2020). Seismic performance of composite joints between prefabricated steel-reinforced concrete columns and steel beams: Experimental study. Bull. Earthq. Eng..

[7] Yang Y., Liao F., Tao Z., Zhang C., Gao X. (2022). Compressive and flexural behavior of prefabricated concrete-filled steel tubular columns with bolted splices. J. Constr. Steel Res..

[8] Liao X., Zhang S., Cao Z., Xiao X. (2021). Seismic performance of a new type of precast shear walls with non-connected vertical distributed reinforcement. J. Build. Eng..

[9] Malla P., Xiong F., Cai G., Xu Y., Larbi A.S., Chen W. (2021). Numerical study on the behaviour of vertical bolted joints for precast concrete wall-based low-rise buildings. J. Build. Eng..

[10] Cao D., Pan Z., Zhang Z., Zeng B. (2023). Study on non-destructive testing method of grouting sleeve compactness with wavelet packet energy ratio change. Constr. Build. Mater..

[11] Hofer L., Zanini M.A., Faleschini F., Toska K., Pellegrino C. (2021). Seismic behavior of precast reinforced concrete column-to-foundation grouted duct connections. Bull. Earthq. Eng..

[12] Guo X., Zhang Y., Xiong Z., Xiang Y. (2016). Load-bearing capacity of occlusive high-strength bolt connections. J. Constr. Steel. Res..

[13] Zhang P., Yu J., Pang Y., Fan J., Guo H., Pan Z. (2021). Experimental study on the mechanical properties of grouted sleeve joint with the fiber-reinforced grouting material. J. Build. Eng..

[14] Zheng G., Kuang Z., Xiao J., Pan Z. (2020). Mechanical performance for defective and repaired grouted sleeve connections under uniaxial and cyclic loadings. Constr. Build. Mater..

[15] Liu J., Li D., Cui X. (2023). Research status and future directions of defect detection in grouted splice sleeves: A review. Constr. Build. Mater..

[16] Wan L., Zhao Y., Yu M., Li N., Sun X. (2022). Experimental study on spatial mechanical properties of grout anchor lap joints. Structures.

[17] Zheng Y., Zhu Z., Guo Z., Liu P. (2019). Behavior and Splice Length of Deformed Bars Lapping in Spirally Confined Grout-Filled Corrugated Duct. Adv. Mater. Sci. Eng..

[18] Ma C., Jiang H., Wang Z. (2019). Experimental investigation of precast RC interior beam-column-slab joints with grouted spiral-confined lap connection. Eng. Struct..

[19] Chen J., Chen X., Ding F., Xiang P., Yang C., Liu Y., Xu F. (2020). Mechanical performance of overlap connections with grout-filled anchor reinforcements in embedded metal corrugated pipe. Arch. Civ. Mech. Eng..

[20] Liu F., Qian H., Zhang Z., Zhang H. (2023). Experimental Study on the Mechanical Properties of Vertical Corrugated Pipe Grout Anchor Connection Joints. Appl. Sci..

[21] Roeder C.W. (2002). Connection Performance for Seismic Design of Steel Moment Frames. J. Struct. Eng..

[22] Luo X., He Y., Chen Q., Chen L. (2023). Study on Joint Connection Performance of an Innovative Tooth Groove Connection and Vertical Reinforcement Lapping in Reserved Hole. Materials.

[23] El-Khier M., Morcous G. (2021). Precast concrete deck-to-girder connection using Ultra-High Performance Concrete (UHPC) shear pockets. Eng. Struct..

[24] Gao Q., Li J.H., Qiu Z.J., Hwang H.J. (2019). Cyclic loading test for interior precast SRC beam-column joints with and without slab. Eng. Struct..

[25] Ge Q., Meng Y.J., Ai J.S., Zuo W.H., Xiong F., Liu Y., Dong N. (2024). Seismic performance of precast concrete sandwich walls with bolt-steel plate connection. Eng. Struct..

[26] Li F.R., Abruzzese D., Milani G., Li S.C. (2022). Influence of internal defects of semi grouted sleeve connections on the seismic performance of precast monolithic concrete columns. J. Build. Eng..

[27] Li D., Liu H. (2019). Detection of sleeve grouting connection defects in fabricated structural joints based on ultrasonic guided waves. Smart. Mater. Struct..

[28] Cao D., Pan Z., Zhen G. (2023). Effects of grouting defects on seismic behavior of full-scale precast reinforced concrete shear wall. J. Build. Eng..

[29] Elsayed M., Nehdi M.L., Provost-Smith D.J., Eissa O.S. (2018). Exploratory investigation of grouted bar-in-duct connections under direct tensile load. Constr. Build. Mater..

[30] Tazarv M., Saiidi M.S. (2017). Design and construction of UHPC-filled duct connections for precast bridge columns in high seismic zones. Struct. Infrastruct. Eng..

[31] Chen J., Zhao C., Ding F., Duan Q., Cao Z., Xu F., Yang C., Lu D., Xiang P. (2020). Mechanical performance of the grouted lapped double reinforcements anchored in embedded corrugated sleeves. Structures.

[32] Wang H., Liang R., Li J., Liu J., Li H., Hu X., Zhu K. (2022). Anchorage performance of grouted corrugated duct connection under monotonic loading: Experimental and numerical investigation. Constr. Build. Mater..

[33] Zhou Y., Ou Y.C., Lee G.C. (2017). Bond-slip responses of stainless reinforcing bars in grouted ducts. Eng. Struct..

[34] Seifi P., Henry R.S., Ingham J.M. (2019). In-plane cyclic testing of precast concrete wall panels with grouted metal duct base connections. Eng. Struct..

[35] Popa V., Papurcu A., Cotofana D., Pascu R. (2015). Experimental testing on emulative connections for precast columns using grouted corrugated steel sleeves. Bull. Earthq. Eng..

[36] Tullini N., Minghini F. (2020). Cyclic test on a precast reinforced concrete column-to-foundation grouted duct connection. Bull. Earthq. Eng..

[37] Tullini N., Minghini F. (2016). Grouted sleeve connections used in precast reinforced concrete construction-Experimental investigation of a column-to-column joint. Eng. Struct..

[38] Aiamsri K., Yaowarat T., Horpibulsuk S., Suddeepong A., Buritatum A., Hiranwatthana K., Nitichote K. (2024). Bonding behavior of lap-spliced reinforcing bars embedded in ultra-high-performance concrete with steel fibers. Dev. Built. Environ..

[39] Alkaysi M., El-Tawil S. (2017). Factors affecting bond development between Ultra High Performance Concrete (UHPC) and steel bar reinforcement. Constr. Build. Mater..

[40] Chen Q., Luo X., Xing M., Li Z. (2023). Shaking table test of fully assembled precast concrete shear wall substructure with tooth groove connection and vertical reinforcement lapping in reserved hole. J. Build. Eng..

[41] (2019). Standard for Test Methods of Concrete Physical and Mechanical Properties.

[42] Gong W., Chen Q., Miao J. (2021). Bond behaviors between copper slag concrete and corroded steel bar after exposure to high temperature. J. Build. Eng..

[43] Jia J., Zhao N., Bai Y., Du X., Yang K., Yang D. (2023). Bond-slip model for rebar mounted to high-strength grouting material. J. Build. Eng..

[44] Zhao J.Q., Zhou L., Ding Y.G., Zhu R.J. (2023). Experiment on anchoring performance of spiral stirrup-corrugated pipe grout splicing. J. Jilin. Univ. (Eng. Tech. Ed.).

[45] Xu G., Chen B., Zeng J., Gao D., Bao H. (2022). Research on critical anchorage length of UHPC-CA and HTRB600E high-strength steel bars. Structures.

[46] Zhao M., Li J., Xie Y., Shen J., Li C. (2024). Experimental study of the bond behavior of 400MPa grade hot-rolled ribbed steel bars in steel fibre reinforced concrete. Sci. Rep..

[47] (2015). Code for Design of Concrete Structures.

[48] (2014). Building Code Requirements for Structural Concrete.

